# Report of the Sanitary Commission of Massachusetts

**Published:** 1851-03

**Authors:** 


ART. V.—*Report of the Sanitary Commission of Massachusetts.* 1850. Boston. 8 vo. pp. 540.

There are few, if any subjects, affecting the temporal welfare of human society, which possess greater importance than the subject of *Public health.* To any intelligent person, after due reflection, this proposition must be almost self-evident. To increase the probabilities of the duration of life, and to diminish the liabilities to disease, are objects which it would be hardly necessary to prove are secondary only to those which concern, more directly, the intellectual, moral, and religious improvement of the race. With the latter, confessedly higher objects, the subject of public health has closer relations than is by many supposed.

The attention which this subject has received from physicians, philanthropists, and legislators, bears an inverse ratio to its intrinsic claims. Its almost utter neglect in times past, would seem quite incredible, did not the history of science exhibit the curious fact, that the tendency of the human mind is to objects remote from human existence. Astronomy was one of the sciences earliest studied. Men were desirous of becoming acquainted with phenomena and laws belonging to other worlds, before they had fairly commenced investigations restricted within terrestrial limits. And as astronomy was one of the earliest, so the science of human life and organization was one of the latest pursuits to be cultivated with assiduity and success. This latter fact affords, in part, an explanation of the remarkable inattention to matters relating to public health. The subject in many of its aspects could not be appreciated while the functions of the organism were very imperfectly understood. The importance of the subject, in its various relations, has, of late years, been progressively advancing in the estimation of intelligent, reflecting portions of society, until it has, at length, become, what it has just claims to be considered, one of the leading subjects of the many which concern the welfare of the human family. In England and France it is emphatically *the* important subject of the day. In this country interest in its behalf, commensurate, in some degree, to its importance, is beginning to be felt. Compared, however, with what has been done, and is now being done, in Europe, the medical profession, and the state, with us, are backward in the sanitary mevements which may be said to be characteristic of the present time.

The subject is one which, although not belonging exclusively to physicians, and indeed is cultivated with distinguished success by others, yet commends itself in an especial manner to members of the medical profession. The medical profession, as well as other branches of society, are open to censure for its past and present neglect. We have been too much engrossed in the investigation of diseases with reference to their pathology, discrimination, and treatment, bestowing far too little consideration upon the questions in how far are the sources of diseases within our control, and how can they be arrested by prophylactic measures rendered applicable to all the individuals composing communities.

With these desultory remarks, which open up various and extensive topics of inquiry, we proceed to invite the attention of our readers to the publication, the title page of which is prefixed. Although not relating to practical medicine, or surgery, we need not amplify on the relevancy of the subject of public health to those inquiries which are peculiarly appropriate to the medical reader; nor will an apology be required for devoting to the subject considerable space in a medical Journal.

In July, 1850, three Commissioners were appointed by the Legislature of Massachusetts, to "prepare, and report to the next General Court, a plan for a Sanitary Survey of the State, embracing a statement of such facts and suggestions as they may think proper to illustrate the subject."

The commissioners selected by the Governor of the State, were Lemuel Shattuck, of Boston, Nath. P. Banks, Jr., of Waltham, and Jehiel Abbott, of Westfield. The name of the chairman, Mr. Shattuck, a member of the legal profession,* has long been identified with the prosecution of statistical investigations relating to health and disease, and it is not doing injustice to the other gentlemen of the committee to state, that the labor of collecting the materials, and preparing the present report, devolved upon him. And we would say here, what we think our readers will infer from the imperfect analysis which will follow, that Mr. S. has produced a work not only highly interesting, but in an eminent degree useful, being well calculated to advance the objects for which it is designed, viz., to enforce the importance of the subject of public health, and to inculcate the means by which the sanitary movement in this country is to be advanced.

By a Sanitary *Survey* is meant, "an examination or Survey of the different parts of the commonwealth—its counties, its towns, and its localities—to ascertain the causes which, favorably or unfavorably, affect the health of its inhabitants."

The following paragraph presents a concise expression of the facts upon which are based the benefits to be expected from such a Survey:—

We believe that the conditions of perfect health, either public or personal, are seldom or never attained, though attainable;—that the average length of human life may be very much extended, and its physical power

* Since this was written we learn that we were incorrectly informed as respects the profession of Mr. S. He is, or has been a merchant.

greatly augmented;—that in every year, within this Commonwealth, thousands of lives are lost which might have been saved;—that tens of thousands of cases of sickness occur, which might have been prevented;—that a vast amount of unnecessarily impaired health, and physical debility, exists among those not actually confined by sickness;—that these preventable evils require an enormous expenditure and loss of money, and impose upon the people unnumbered and immeasurable calamities, pecuniary, social, physical, mental, and moral, which might be avoided;—that means exist, within our reach, for their mitigation or removal; and that measures for their prevention will effect infinitely more, than remedies for the cure of disease.

The relative value heretofore attached by Legislators to scientific investigations relating to public health is significantly shown in the facts contained in a passage given in a note, which we quote, with the remark that facts quite as significant, if not much more so, might be cited from the legislative proceedings of the State of New York within the few past years.

The valuable Reports of the Commissions heretofore existing in Massachusetts, are of considerable length. That on *Insects* contains 460 pages; that on *Invertebrata*, 374 pages; that on *Fishes*, *Reptiles*, and *Birds*, 416 pages; and that on *Trees* and *Shrubs*, 547 pages; besides illustrative plates in each. The first of these reports has been ordered to be reprinted this year. It would be reasonable to suppose that MAN was entitled to a consideration equal to either of these subjects.

The first part of the work is devoted to the past history, and the present condition of the Sanitary movement. We cannot condense all the valuable information contained in the one hundred pages occupied with these topics. The historical and other details pertaining to the Sanitary movement in Great Britain are chiefly dwelt upon. The prime actor in this movement in that country was Edwin Chadwick, Esq., barrister-at-law. It was chiefly through the able writings of this gentleman, in 1828, that the attention of Parliament was directed to the subject. The publications of Dr. T. Southwood Smith were also instrumental in bringing about this result. In 1832 was appointed a commission to "inquire into the practical operation of the laws for the relief of the poor in England and Wales;" and in 1833 another, "to consider and report on the general state of parochial registers, and on a general registration of births, baptisms, marriages, deaths and burials, in England and Wales." In consequence of the information contained in the latter report, "an act for the registration of births, marriages, and deaths, in England and Wales," was passed in 1836, and went into operation in 1837, a central office being established in London, presided over by an officer styled the *Registrar-General.* The quarterly reports which have since emanated from the office of the Registrar-General, are of very great value. The author of the Massachusetts Report gives a compilation from the appendix of the ninth annual report of the Registrar-General, to show the rate of mortality among four different populations in England, to wit, 1, the whole of England; 2, one of the most healthy districts; 3, one of the most unhealthy districts; 4, London. Now, a single item of the several points of disparity between these populations, obtained by registration, suffices to illustrate the great importance of vital statistics. In the most healthy districts of England 4.123 per cent. of the males die under five years of age; while in the most unhealthy 14.372 per cent. die in the same age! This is but one point of comparison. Other differences not less striking appear from the tables. Such facts show a preponderance of morbid influences commensurate with the increased ratio of mortality in certain sections. Then the important questions arise, whence this preponderance? what are the causes of disease which occasion in one section a degree of mortality nearly four times greater than in another section? These questions are to be answered by facts developed in the prosecution of Sanitary Surveys; and upon the conclusions thus arrived at, are to be based those measures of sanitary improvement which will diminish the operation of morbid influences, reduce the rate of mortality, and increase all the desirable results flowing from augmented health and prolonged life.

During the last twelve years several valuable publications, in addition to the reports of the Registrar-General, have emanated from the British press. Among those deserving particular mention are the following: statistical reports of Maj. Tulloch on the sickness, mortality, and invaliding among the British troops stationed in different quarters of the globe; statistical reports on the health of the Navy, by Dr. John Wilson; report of a select committee appointed by the house of commons, on the "circumstances affecting the health of the inhabitants of large towns, and populous districts; reports upon the employment of the children of the poorer classes in mines and collieries, etc.; report of the poor law commissioners on the training of pauper children; report on the prevalence of certain physical causes of fever in the metropolis, by Drs. Arnott and Kay; several reports on the physical causes of sickness and mortality to which the poor are exposed, and on the prevalence of fever, by Dr. Southwood Smith; reports on interments in towns, by E. Chadwick, Esq. Several able papers have also appeared in leading periodical reviews. Mr. Shattuck sums up the conclusions to which he is led from an analysis of the documents and works just referred to, in the following propositions:

1. *It is proved* that there die annually, in each 100 of the population, of the whole of England, 2.27; of the most healthy district, 1.53; and of the most unhealthy district, 3.58. And that the living to one death are, in these districts, respectively, 44, 65, and 27.

2. *It is proved* "that the various forms of epidemic, contagious, and other diseases, caused, or aggravated, or propagated, by atmospheric impurities, produced by decomposing animal or vegetable substances, by damp and filth, and close and over crowded dwellings, prevail amongst the population in every part of the kingdom, whether dwelling in separate houses, in rural villages, in small towns, or in the large towns, as they have been found to prevail in the lowest district of the metropolis."

3. *It is proved* that disease and mortality fall more heavily upon those who live in large towns and populous places, than in the country districts, and particularly upon those who live in narrow streets, confined courts, damp dwellings, close chambers, cellars, undrained, unventilated, and uncleansed; and affect most severely the infantile portion of the population, and the heads of families between twenty and thirty years of age.

4. *It is proved* that, in such situations, the average duration of life is five to twenty-five years less than it might otherwise be; and that, during this curtailed period of existence, the working power of those who live, and their capacity for enjoyment, are greatly diminished by a constant depression of health and spirits, and by the active attacks of fever, cholera, scrofula, and consumption.

5. *It is proved* "that such diseases, wherever their attacks are frequent, are always found in connection with the physical circumstances above specified; and that where these circumstances are removed by drainage, proper cleansing, better ventilation, and other means of diminishing atmospheric impurity, the frequency and intensity of such diseases are abated; and where the removal of the noxious agencies, and other causes of disease, appears to be complete, such diseases almost entirely disappear."

6. *It is proved* that the annual mortality might be reduced, in the whole kingdom, from 2.27 per cent., or 1 in 44, to less than two per cent., or 1 in 50; and in all large towns, as low as that general average.

7. *It is proved* that this unnecessary excess of mortality above 2 per cent., occasions an annual loss of more than 50,000 lives in the United Kingdom,—"greater than the loss from death or wounds in any wars in which the country has been engaged in modern times;" and that the causes of these unnecessary deaths occasion at least twenty cases of unnecessary sickness, on the average, to each death, or one million cases annually, which might have been prevented.

8. *It is proved* that of the 43,000 cases of widowhood, and 112,000 cases of destitute orphanage, relieved from the poor rates of England and Wales alone, the greater proportion of deaths of the heads of families occurred from specified removable causes; and that the average of their ages was under forty-five years, or thirteen years below the natural probability of life, as shown by experience.

9. *It is proved* that the preventable causes of disease, and the unnecessary mortality, impose upon the people immense pecuniary burdens which might be avoided.

10. *It is proved* that the younger population, bred up under noxious physical agencies, is inferior in physical organization and general health to a population preserved from such agencies; and that these adverse circumstances tend to produce an adult population, short-lived, improvident, reckless, intemperate, immoral, and with excessive desires for sensual gratifications.

Having given an historical sketch of what has been done in other countries toward the sanitary movement, Mr. Shattuck next enters upon the consideration of this movement at home. He has consulted the past history of the country in order to collect therefrom information respecting public health, in times gone by. Many interesting and curious items are presented under this head, but in number and character they are far from being satisfactory for purposes of scientific investigation. On the meagreness of historical annals with respect to this subject, even in modern days, he thus remarks:

For the last forty years, notwithstanding the mass of medical literature that has been published, less definite information has been obtained concerning epidemics than in the previous periods. The almost entire neglect of records, prior to the adoption of the registration system, renders it difficult to give any thing approximating to an accurate view of the subject. If a careful examination were made into the history of each town, many important facts might be gathered. But it is curious and lamentable to observe, in looking over our published local histories, how little attention has been paid to this matter. *The History of the Health of the People* should be regarded as the most important part of history, yet it has generally been considered unworthy of notice, or, if noticed at all, merely among the incidental matters of little consequence.

The sanitary movement can only be said to have as yet commenced in this country, although, during the short spaee of time that any attention has been directed to the subject, many valuable contributions have been made. The Transactions of the American Medical Association already contain some interesting and useful papers furnished by Mr. Shattuck and others. It is obvious that the movement cannot advance without a registration of births, marriages, and deaths. Legislation has lately provided for such a registration in Massachusetts, which is now in successful operation. We are not aware that this is the case in any other State, but on this point we do not profess to have accurate information. An act was passed a few years ago providing for such a registration in this State, but from some defects in details it did not succeed, and is now, so far as we know, to all intents inoperative. To secure this object in all the States is a great desideratum. This, indeed, is the initiative in the progress of the sanitary movement—it is the first step. Regarding it in this light the Am. Med. Association at one of its first meetings urged upon the medical societies of the different States to endeavor to enlist the interest of legislators in the subject, and procure effective legal enactments.

To illustrate the importance of the results to be deduced from an examination of the statistics obtained by means of registration reports, Mr. Shattuck gives a statement of the rate of mortality among the inhabitants of Boston for 1830, 1840, and 1845; and of an interior country town in Mass. for 1830. The rate of mortality of different ages from 5 years and under, up to 70 and over, is given in this statement in a tabular form. By a glance at the latter the reader is able to determine the liability to death at different ages, and of all ages collectively, in the different places specified. For all ages the average rate of mortality for the space of nine years was 2.53 per cent. In the towns in the country it was 1.49 per cent. In Boston the mortality under 5 years of age was 9 per cent.; in the country only 3.05 per cent. It is evident from these specimens of the facts thus elicited, that statistics from all parts of the United States would open a wide field for comparisons, possessing great value, as well as interest. Another table compiled by Mr. Shattuck from the registration reports for five consecutive years, shows the influence of the seasons, in Mass, on mortality. The deaths during each months in the five years, for different ages, are given in this statement. The results are that the summer quarter is attended with the most fatality; next the autumnal; next winter, and last the spring. August and September are the most unhealthy months in Boston, and October the most unhealthy month in the country. The fatal diseases during these months in the city affect the digestive organs, and in the country the occasion of the greatest fatality in October is the prevalence of fevers in this month.

Statistics from registration reports are adduced to exhibit the influences on health and longevity derived from *occupation*, and domestic condition. From these it appears that *farmers* are the longest lived; next to these, *hatters;* next, *coopers;* next, *clergymen;* next, *lawyers;* next, *physicians*, etc. *Stone cutters, shoemakers, painters*, and *laborers* are low in the scale, but mechanics and printers are the shortest lived. The average length of married life is found to be, of men, 28.90 years, and of women, 22.16. The average length of the period of widowhood is, of men, 19.33, and of women, 28.90 years. The average age at which men marry, for the first time, in Mass., is 25.71 years; and women, 26.61 years.

To illustrate the influence of disease, which is the most important test of the sanitary condition of a State, the author presents a table containing the number of deaths in Boston by each known cause from 1811 to 1849 inclusive, a period of 39 years. This table furnishes the requisite data for judging of the comparative prevalence of different diseases at Boston during the time mentioned. The table also contains the number of deaths from known diseases in the remainder of the State, i. e. exclusive of Boston, during the last seven years, the period covered by the registration reports provided for by the laws of Massachusetts.

The fatality from diseases classed under the denomination *zymotic*, i. e. those which are due to epidemic, endemic or contagious causes, as appears from this table, has been constantly increasing during the last 36 years. As these diseases constitute the great index of public health, this fact shows that the sanitary condition has been progressively deteriorating. Of the individual zymotic diseases, dysentery, cholera infantum, scarlatina, and small pox are those which have especially increased. Deaths from small pox have increased from 0.8 to 1.34 per cent. In the country 9.09 per cent. of all the deaths are from Continued fever. Deaths from hydrocephalus have nearly doubled in Boston within the last 30 years. The results of an analysis of the latter with reference to the effects of season, age, and sex, upon pulmonary consumption, are stated, which we must omit. Finally, the author concludes this division of the subject by the enunciation of several conclusions which we copy:—

1. *It is proved* that there is a great difference, in this State, in the longevity of people living in different places and under different circumstances.

2. *It is proved* that causes exist in Massachusetts, as in England, to produce premature and preventable deaths, and hence unnecessary and preventable sickness; and that these causes are active in all the agricultural towns, but press most heavily upon cities and populous villages.

3. *It is proved* that measures—legislative, social and personal—do not at present exist, or are not so fully applied, as they might be, by the people, for the prevention, mitigation, or removal, of the causes of disease and death.

4. *It is proved* that the people of this State are constantly liable to typhus, cholera, dysentery, scarlatina, small-pox, and the other great epidemics; and to consumption, and the other fatal diseases, which destroy so many of the human race in other parts of the world.

5. *It is fully proved* that the active causes of disease and death are increasing among us, and that the average duration of life is not as great now as it was forty or fifty years ago.

We are fully aware that the general opinion does not coincide with this fact, and that a directly opposite one has been expressed. It has been frequently said, that, owing to the different modes of living, the increased medical skill, and other causes, diseases have been ameliorated, and the average length of human life has been extended; and particularly within the last fifty years. We have long thought differently, especially in regard to the more recent periods of our history. Those who make this assertion seem to rely upon imperfect or uncertain data to support their opinion. Statistical observations of the living and the dead, gathered in ancient times, should be taken with great caution as comparative tests. Ten years since, it was said that "the average value of life is not as great as it was twenty years ago; that it was at its maximum in 1810 to 1820; and that it has since decreased."* Subsequent investigations have fully established the correctness of this statement.

It is udoubtedly true, that in many things society has improved; that medical skill in the cure of disease has greatly increased; and that some diseases are not as fatal as formerly, or are now better understood and controlled. But while all this may be true, it is no less true that the active causes of disease have increased faster than the appliances for their prevention and cure; that new diseases, or old ones in a new and modified form, equally fatal and uncontrollable, have appeared; and that sickness and death advance more rapidly than the improvements devised to arrest them.

These statements, concerning the decreasing vital energies of man, are confirmed by recent investigations in England.

In the third division of the Report Mr. Shattuck presents a *plan for a Sanitary Survey of the State.* The plan embraces measures to be regulated and controlled, *first* by the legislative authority of the State, and, *second,* by social organization and personal action. The several measures proposed are stated in a series of articles each of which is considered by Mr. S. at more or less length. This portion of the report covers an hundred and thirty-seven pages. Our limits will only allow us to present a list of the more important measures, omitting the considerations which the author offers respecting their applicability, importance, and the means by which they are to be enforced. As the reader may imagine, we are thus obliged to forego any account of much valuable information concerning the various topics involved in the subject of public health. We shall give the more important recommendations in succession, but without the division into separate articles which is adopted in the Report.

A revision of the laws of the State relating to Public health is advised, and a new act to be passed in their stead.

The general execution of the laws of the State relating to the enumeration, the vital statistics, and the public health of the inhabitants, should be entrusted to a General Board of Health.

This board should be composed of two physicians, one counsellor at law, one chemist or natural philosopher, one civil engineer, and two persons of other professions or occupations; all properly qualified for the office by their talents, their education, their experience, and their wisdom.

A secretary, to be appointed by the Board, should be required to devote

---

* American Journal of Medicine for 1841, Vol. 1, p. 382.

his whole time and energies to the discharge of the duties of his office, and secure a proper salary for his services. A LOCAL BOARD OF HEALTH, should be appointed in every city and town, charged with the particular execution of the laws of the State, and the municipal ordinances and regulations relating to public health, within their respective jurisdictions; and these local boards should appoint a secretary, and, if occasion require, a surveyor and health officer.

The local boards should endeavor to ascertain, with as much correctness as possible, the circumstances of the cities and towns, and of the inhabitants under their jurisdiction; and issue such local sanitary orders, and make such regulations as are best adapted to their circumstances, resorting to compulsory process only when the public good requires it.

Appropriations should be annually made by the State for the purchase of books for the use of the General board of health; and by each city and town for the purchase of books for the use of each local board of health.

Each local board of health should be required to make a written report annually to the town, concerning its sanitary condition, during the next preceding year, and to transmit a written or printed copy to the next general board of health.

Successive enumerations of the inhabitants of the State should be so made, abstracted, and published, that the most useful and desirable information concerning the population may be ascertained.

The laws relating to the public registration of births marriages and deaths, should be perfected, and carried into effect in every city and town of the State.

Provisions should be made for obtaining observations of the atmospheric phenomena, on a systematic and uniform plan, at different stations in the commonwealth.

An uniform nomenclature for the causes of death, and for the causes of disease, should be used in all sanitary investigations and regulations.

In laying out new towns and villages, and in extending those already laid out, ample provision should be made for a supply, in purity and abundance, of light, air, and water; for drainage and sewerage, for paving, and for cleanliness.

In erecting school houses, churches, and other public buildings, health should be regarded in their site, heating apparatus, and ventilation; and before erecting any new dwelling house, manufactory, or other building, for personal accommodation, either as lodging house, or place of business, the owner or builder should be required to give notice to the local board of health of his intention, and of the sanitary arrangements he proposes to adopt. The local boards of health, also, should endeavor to prevent or mitigate the sanitary evils arising frcm overcrowded lodging houses and cellar dwellings.

Open spaces should be reserved in cities and villages for public walks; wide streets should be laid out, and both should be ornamented with trees. Special sanitary surveys of particular cities, towns, and localities, to be made, from time to time, under the direction of the General Board of Health.

Local boards of health, and other persons interested, should endeavor to to ascertain, by exact observation, the effect of mill-ponds, and other collections and steams of water, and of their rise and fall, upon the health of the neighboring inhabitants.

The local boards should provide for periodical house-to-house visitation, for the prevention of epidemic diseases, and for other sanitary purposes. The importance of this measure in epidemic cholera is exemplified in a striking manner by the following results of a general order for its introduction into London in 1849, for the first 52 days after its adoption:—

| | |
|---|---|
| Diarrhœa, cases discovered, . . . | 43,127. |
| Rice water purging discovered, . . . | 976. |
| Cholera discovered, . . . . . . | 779. |
| Passed into cholera after treatment, . . | 52. |

The author adds, "had it not been for these visitations very many more of these cases would have terminated in cholera and death. What facts can more forcibly illustrate the utility of preventive measures."

Measures should be taken to ascertain the amount of sickness suffered in different localities; and among persons of different classes, professions, and occupations.

Measures should be taken to ascertain the amount of sickness suffered, among the scholars who attend the public schools and other seminaries of learning in the commonwealth.

Every city and town should be required to provide means for the periodical vaccination of the inhabitants.

The causes of consumption and the circumstances under which it occurs, should be made the subject of particular observation and investigation.

Measures should be taken to prevent or mitigate the sanitary evils arising from the use of intoxicating drinks, and from haunts of dissipation.

The General management of cemeteries and other places of burial, and of the interment of the dead, should be regulated by the local boards of health.

Measures should be adopted for preventing or mitigating the sanitary evils arising from foreign emigration.

The foregoing measures are those to be adopted under state and municipal authority. The following are to be carried into effect without any special legislative authority, and are therefore distinguished as social and personal measures:—

A sanitary association should be formed in every city and town for the purpose of collecting and diffusing information relating to public and personal health.

Tenements for the better accommodation of the poor should be erected in cities and villages.

Public bathing houses and work houses should be established in all cities and villages.

The refuse and sewerage of cities and towns should be collected and applied to purposes of agriculture; and the smoke nuisance should be prevented as far as practicable.

The sanitary effects of patent medicines and other nostrums and secret remedies should be observed; and medical compounds advertised for sale be avoided unless the material of which they are composed be known, or unless manufactured and sold by a person of known honesty and integrity.

The local boards of health and others interested should endeavor to prevent the sale and use of unwholesome, spurious, and adulterated articles, dangerous to public health, designed for food, drink, or medicine.

Institutions should be formed to educate and qualify females to be nurses of the sick.

Persons should be specially educated in sanitary science, as preventive advisers, as well as curative advisers.

Physicians should keep records of cases professionally attended.

Clergymen of all religious denominations should make public health the subject of one or more discourses annually before their congregations.

Having submitted the various measures involved in a complete sanitary reform, Mr. Shattuck next considers the reasons for approving of the plan recommended, and meets several objections to it which, it may be anticipated, some of his readers may offer. He then gives a bill for enactment by the legislature, embodying provisions for carrying the plan into execution.

This concludes the Report proper, embracing 321 pages. The publication, however, contains an appendix of about 225 pages. The latter contains all the acts relating to public health that have been passed by the state of Massachusetts; sanitary surveys of several towns in the State, and several miscellaneous papers relating to the subject of health, all of which possess importance and interest.

We have devoted considerable space to an analytical notice of this work, but not more than is due to its merits, as well as to the subject of which it treats. In our view it is the most valuable publication which has, for some time past, emanated from the American press. The medical profession, and the public, are under no small degree of obligation to Mr. Shattuck for his labors in behalf of public health, and we sincerely hope that the time is not far distant when he will be repaid by the satisfaction of seeing his important recommendations adopted, not only in Massachusetts, but in every State of the Union.

To effect an end so desirable, medical practitioners must become more generally imbued with a sense of the practical benefits which are to flow from its attainment. It belongs to them to give direction and impetus to public opinion as the basis of intelligent and efficient legislation on the subject. There are two points of view in which the sanitary movement appeals most forcibly to the physician for support and encouragement. *First,* it proposes to increase the sum of life and health. We need not look farther than this, and inquire in how far national health, and individual happiness, are to be enhanced by such a result. The object is sufficient in itself. More health and a longer average of life are attainable—this is enough for the physician, for these are what he chiefly aims at in the discharge of his professional duties. He strives to increase the sum of life and health by curing diseases. should he not then sympathize in efforts to secure the same humane purposes by measures calculated to develop the ability to resist and surround disease, and by warding off the causes of disease? As we have already remarked, the latter have, undoubtedly, received a share of attention from medical philosophers disproportionate to their relative consequence. The grand objects have been to mitigate diseases after their development: to study their pathological characters, the methods of discriminating them, and their therapeutical indications. These are subjects of vast importance, but yet of not greater importance to the welfare of society than the prevention of disease. And here we may cite a fact with respect to the latter which will serve to enforce its comparative claims: The progress of the scientific investigation of diseases is not attended by corresponding success in their therapeutical management. This fact must be admitted. We do not mean to assert that the therapeutical branch of medical science has not advanced, and is not constantly advancing. Far from this. But it will not be denied that discoveries in this branch have been less brilliant than in morbid anatomy, pathology, and diagnosis. This fact should serve to turn our attention to sanitary inquiries, to ascertain if, in that way, we cannot realize more fully the ultimate aim of all medical science—to wit—the increase of life and health. Moreover, the progress of our knowledge of diseases teaches us that we cannot expect to cure them, in proportion as we succeed in their investigation. The lesson is, indeed, sometimes quite the reverse of this, viz., that if we expect to diminish the mortality from some diseases which are the most frequent in their occurrence, as well as the most fatal in their character, we must ascertain, and subject to our control their causes—in other words the reliance is on prevention rather than cure. Tuberculosis may be mentioned as a striking illustration of the truth of this remark.

This leads us to the *second* point of view in which the sanitary movement appeals to the physician. It proposes means of ascertaining and controling the causes of disease—it aims at prevention. As respects etiology, the statistical facts developed by registration reports seem to us to be of very great importance. By determining the value of life as affected by differences of residence, occupation, etc., we are enabled to appreciate the different degrees of morbific agency due to these extrinsic, and, in a measure, controllable circumstances; and by establishing relations between these circumstances and the prevalence of particular diseases, the sources whence the latter originate are made apparent. Suppose, for an example, that we discover a particular occupation or locality to be more prejudicial to life and health than other localities and occupations, the inquiry at once arises, what are the morbid agencies incident to this occupation or locality which account for such a disparity, and how do they operate upon the organism? Investigation follows this inquiry, and is thus excited and directed by the conclusions deduced from statistics. Again, we discover from the registration tables that certain diseases prevail among a certain class, in certain sections, or in certain seasons; we are then led to seek for the etiological causes therein involved. Much information has already been evolved in this way, but much more remains to be developed as the accumulation of data increases, in consequence not only of a longer period for their collection, but of a more general adoption of the system. In thus attaching great value to sanitary statistics we do not overlook the fact that we are liable to error from this source, and that deductions are to be made with great care and circumspection. Figures are by no means infallible. They constitute but a means of disclosing truth, and, like all other potent instruments, are to be managed with caution and skill. In their application to the study of health, as well as disease, they are seldom in themselves adequate to compass the objects desired, but they open the way, and point to the right direction. Statistical results do not constitute the final objects of our pursuit,, but they furnish the *bee lines* which direct our steps in the way of successful investigation.

